# Body Self-Perception After Breast Reconstruction in Young Female Patients Affected by Poland Syndrome

**DOI:** 10.1007/s00266-022-02859-x

**Published:** 2022-03-25

**Authors:** Ilaria Baldelli, Monica Zena, Monica Vappiani, Valeria Berrino, Marco Bruzzone, Maria Lucia Mangialardi, Edoardo Raposio

**Affiliations:** 1 Department of Surgical Sciences and Integrated Diagnostics (DISC), University of Genoa, Viale Benedetto XV, 6, 2nd Floor, 16132 Genoa, Italy; 2 Plastic and Reconstructive Surgery Clinic, Policlinico San Martino Hospital, pad. 15, L.go R. Benzi 10, 16132 Genoa, Italy; 3 Unit of Clinical epidemiology, Policlinico San Martino Hospital, L.go R. Benzi 10, 16132 Genoa, Italy

**Keywords:** Poland syndrome, Breast asymmetry, Breast reconstruction, Body image, Body uneasiness, Quality of life

## Abstract

**Background:**

Cosmetic and social aspects of breast anomalies in Poland syndrome are not negligible. Early diagnosis and appropriate therapeutic timing may have a positive impact on quality of life.

**Methods:**

Females affected by Poland syndrome, who had breast reconstruction between 2014 and 2018, were asked to complete the Body Uneasiness Test and the postoperative Breast-Q. Correlation between scores was evaluated. Correlation between scores was statistically evaluated.

**Results:**

Thirty patients who had completed breast reconstruction at 20.9 ± 6.5 yo fulfilled the questionnaires at the average age of 26.5 ± 8.1 yo. BUT scores were similar to healthy population considering different age groups, with the exception of Compulsive Self-Monitoring subscale for 16–17-year age group. A correlation between Depersonalization and “Thighs” and “Legs” was present. On average, satisfaction with breast resulted 79.1%, satisfaction with surgical outcome was 94.9%, psychosocial well-being was 78.5%, sexual well-being was 75.3%, and relative physical well-being in chest and upper body was 36.9%. Global Uneasiness, Avoidance, Weight Phobia, Body Image Concern and Depersonalization were significantly correlated with lower psychosocial well-being. Avoidance was significantly correlated with lower sexual well-being.

**Conclusions:**

Breast reconstruction in Poland syndrome can help to improve quality of life. However, general body uneasiness can affect satisfaction with the final result.

**Level of Evidence IV:**

This journal requires that authors assign a level of evidence to each article. For a full description of these Evidence-Based Medicine ratings, please refer to the Table of Contents or the online Instructions to Authors http://www.springer.com/00266.

## Introduction

Poland syndrome (PS) is a rare congenital disease characterized by the unilateral, partial or complete, lack of the pectoralis major muscle. Generally, this main criterion is associated with various ipsilateral deformities affecting soft and skeletal tissues of thorax and upper limb [1]. Some type of breast anomaly is always present, and it may consist of a simple asymmetry in shape and volume up to the complete absence of the breast on the affected side. Rib cage deformities can vary from mild forms of asymmetric pectus excavatum/carinatum and/or underdevelopment of multiple ribs until complex sternal deformities and/or absence of multiple costal arcs [2]. Hand and upper limb anomalies are reported in about 50% of patients and are generally characterized by symbrachydactyly, more rarely consisting in absent or non-functioning fingers [3, 4].

Reconstructive thoracic surgery is mandatory when functional impairment is present. Sometimes, a thoracoplasty can facilitate implant-based breast reconstruction when severe chest wall deformities are present. However, most patients are only affected by a main aesthetic problem, characterized by the lack of the anterior axillary pillar and a subclavicular hollow in case of agenesis of the pectoralis major muscle, as well as breast asymmetry and dislocation of the nipple–areola complex. Cosmetic and social aspects of this pathology are not negligible, especially in female patients. The social and cultural context, in fact, plays a fundamental role in the construction of main reference models to which people are inspired for the evaluation of their body image [5]. These models, often unattainable because close to perfection, can become, especially for girls, points of reference from which to judge their own value, leading to a strong personal dissatisfaction [6]. Body dissatisfaction refers to discontent with one’s own physical characteristics, such as weight, body shape, facial features, etc. [7]. Being unhappy with a part of the body does not necessarily mean being dissatisfied with the whole self-image. However, a single defect can be perceived as an element that disturbs the overall physical appearance [8]. In 90’s, the body image disorder was described as a “persistent state of dissatisfaction, worry and discomfort related to an aspect of appearance…” associated with “a certain degree of uneasiness in social relations, social activities or work functioning”[9]. Body image disorders can occur with frequent control of weight and body parts [10] or avoidance behavior, such as wearing loose clothing or avoiding going to particular places such as swimming pools in order to avoid comparisons with other people [11].

Considering that patients affected by Poland syndrome seem to experience maximum discomfort during adolescence [12], early diagnosis and timing in therapeutic approach may have an extremely positive impact on body image and quality of life of young patients [13].

This study aims to investigate body uneasiness, satisfaction with surgery and quality of life of young female patients with Poland syndrome after breast reconstruction.

## Patients and Methods

All participants received a detailed explanation of the study design and a written informed consent was obtained from all respondents, according to the guidelines provided in the current version of the Declaration of Helsinki. Institution Review Board was obtained. Female patients affected by Poland syndrome who had received breast reconstruction in the period 2014–2018 at Plastic and Reconstructive Surgery Clinic Plastic and Reconstructive Surgery Clinic of San Martino Hospital in Genoa were included in this retrospective observational study. Inclusion criteria were female sex, age ≥ 14 years old, breast reconstruction with implant and/or autologous fat graft. Exclusion criterion was breast reconstruction based on flaps.

Patients’ demographic data (age and BMI) and clinical data (laterality of the syndrome, thorax and hand deformity, age at first surgery, number and type of surgeries, age at the end of surgery, complications and surgical revisions) were collected. TBN Classification [2] was used to classify each patient’s thorax deformity; it is based on the following parameters: T–thoracic: from T1 (isolated pectoralis muscle defect) to T4 (pectoralis muscle, sternum and rib defect); B—breast (for females only): B1 (breast hypoplasia) or B2 (breast aplasia); N–nipple–areola complex: from N1 (hypoplasia with dislocation of < 2 cm) to N3 (absence of NAC). Patient’ demographic and clinical data are shown in Table 1.

**Table 1:** Patients’ demographic and clinical data

Patients’ characteristics	Mean ± ds
Age at questionnaire	26.5 ± 8.1
Age at beginning of reconstruction	19.8 ± 6.7
Age at end of reconstruction	20.9 ± 6.5
BMI	22.4 ± 3.3

Clinical data	No.	%
*Reconstructive pathway period*		
≤ 20 yo	19	63.3
> 20 yo	11	36.7
*Side*		
Right	20	66.7
Left	10	33.3
*Hand deformity*		
No	23	76.7
Yes	7	23.3
*Chest deformity*		
No	19	63.3
Yes	11	36.7
*Thoracoplasty*		
No	27	90.0
Yes	3	10.0
*Cutaneous expansion*		
No	3	10.0
Yes	27	90.0
*Contralateral surgery*		
Breast augmentation	9	30.0
Mastopexy	5	16.7
Breast reduction	4	13.3
*Complications*		
Physiological	8	26.7
No	17	56.7
True	5	16.7
*Surgical revision*		
No	18	60.0
Early	9	30.0
Late	3	10.0
*Weight change > 10 kg*		
No	27	90.0
Yes	3	10.0

Patients were recalled in 2019 and asked to complete 2 self-assessment questionnaires:

- The Body Uneasiness Test (BUT) questionnaire is a self-administered test for the assessment of body image disorders [14]. In particular, it consists of two sections: BUT-A, with 34 items regarding experiences inherent to one’s own body, and BUT-B, consisting of the evaluation of hatred toward a list of 37 body parts and body functions. Scores range from 0 (= never) to 5 (= always). Higher scores mean greater body uneasiness. They were analyzed considering the total score (Global Severity Index, GSI) and several subscales [weight phobia (WP), body image concerns (BIC), avoidance (A), compulsive self-monitoring (CSM) and detachment and estrangement feelings toward one’s own body (= depersonalization, D)]. In addition, items from BUT-B with scores of 1 or higher were considered individually to identify worries about specified body parts, shapes and functions.
- The BREAST-Q version 1.0 Reconstruction Module Postoperative Scales is a self-assessment questionnaire developed to measure the quality of life (psychosocial well-being, sexual well-being and physical well-being) and satisfaction of patients who underwent breast surgery (satisfaction with breasts and satisfaction with outcome) [15].

## Surgical Technique

**Fig. 1:**
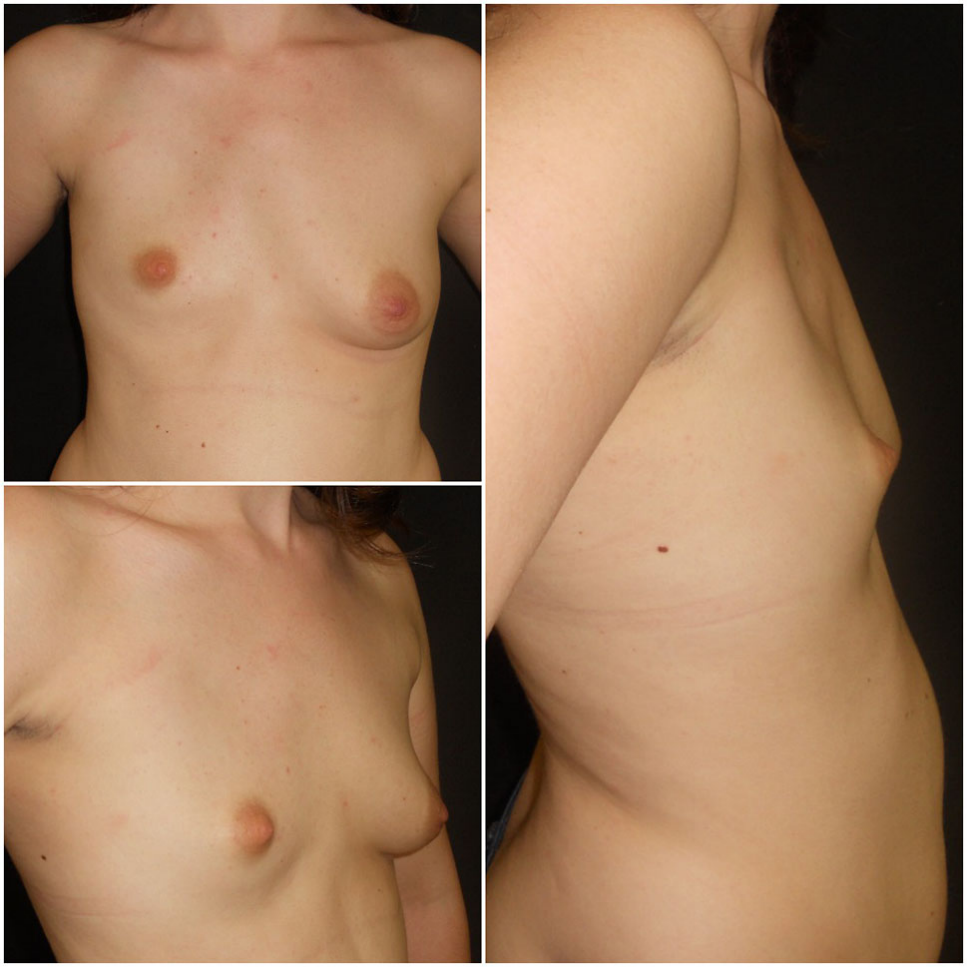
Young woman with T1B1N2 malformation

Breast reconstruction consisted in one or more surgical steps depending on nipple displacement, asymmetry of breast volume and shape, presence of the clavicle portion of the pectoralis major muscle and patient’s expectation and needs [16] (Fig. 1).

In particular, a series of autologous fat graft were used to reconstruct the axillary pillar and increase the thickness of the subcutaneous tissue of the affected side.

**Fig. 2:**
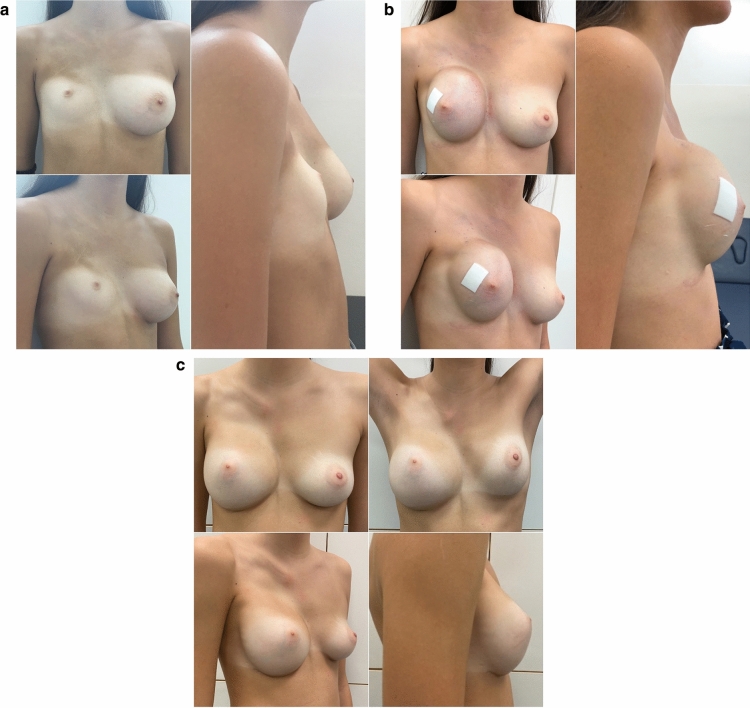
Girl (15yo) with T2B1N2 severe malformation. **a** Preoperative situation. **b** Skin expansion phase. Fat grafting (80 cc) was first performed to restore the subcutaneous tissue of the chest. A tissue expander (Allergan^®^ 133*Plus*SX 400 cm^3^) was then inserted in an upside-down fashion in order to achieve the maximum expansion in the upper poles. An 8×10 cm patch of human-derived acellular dermal matrix was used to provide more support to the pocket medially. **c** 2 years postoperative outcome after removal of the expander, positioning of the definitive implant (Mentor^®^ CPG 331 425 cc) and simultaneous fat grafting (30 cc). The patient was informed about the possibility to achieve a better symmetry through contralateral augmentation mammoplasty, but she is happy with the result obtained so far and does not feel the need of augmentation of the unaffected breast

Nipple displacement was the main parameter to assess the need for expansion of the skin envelope before restore breast volume with a subcutaneous implant or fat grafting. In general, patients with upward and/or medial dislocation nipple dislocation greater than 2 cm compared to the healthy side were candidates for customized tissue expansion to achieve a satisfactory breast symmetry (Figure 2).

Contralateral breast volume reshape/reduction was frequent required, and breast augmentation was sometimes useful or required by patients who desire an overall increase in breast volume.

## Statistical Analysis

For continuous variables, results are presented as mean (SD). The unpaired Student’s t test was used for between-group comparisons. Fisher’s exact test was used to assess differences in the prevalence of non-continuous variables between the two groups. A two-tailed *p*-value < 0.05 was considered statistically significant.

Pearson correlation coefficient was calculated, and linear regression models were performed to evaluate correlation between BREAST-Q questionnaire and BUT-A scores.

Statistical analysis was done using the software Stata/SE 13.1 (StataCorp LP, Texas, USA).

## Results

Thirty female patients affected by PS who had completed their surgical process performed by the same surgeon filled in the questionnaires at the average age of 26.5 ± 8.1 years (Table 1). The right side was affected in 20 patients, and the left one in 10 patients. About half of the patients (*n* = 14) showed a thoracic malformation corresponding to T1B1N2 according to Romanini et al^2^; the absence of clinically evident thoracic cage malformations associated with unilateral mammary hypoplasia and dislocation of the nipple–areola complex more than 2 centimeters with respect to the healthy side (Figure 1). Malformations (symbrachydactyly) of the ipsilateral hand were present in 7 patients (23.3%); syndactyly was corrected in childhood in all of them. Moreover, 11 patients (36.7%) suffered from a malformation of the rib cage of variable severity; however, only 3 patients underwent thoracoplasty before breast reconstruction. The average age at the beginning of the reconstructive process was 19.8 ± 6.7 years, while the average age corresponding to its end was 20.9 ± 6.5 years, with an average time of 385.0 ± 244.1 days to complete the surgical path that was managed before age of 20 in 63.3% of patients (*n* = 19).

Reconstructive protocol included autologous adipose grafting, cutaneous expansion and insertion of a subcutaneous breast implant in 26 patients; 3 patients underwent a direct-to-implant breast reconstruction without skin expansion; and 1 patient had 2 sessions of fat grafting to increase the volume of the affected breast. The number of surgeries required to complete the reconstruction was 3.1 ± 1.1, excluding surgical revisions. Contralateral breast underwent adjustment in 18 patients (60%).

Complications occurred in 5 cases (16.7%), generally referred to implant dislocation and/or capsular contracture, while, in a further 8 patients (26.7%), recurrence of the asymmetry was related to physiological changes (weight changes, growth, pregnancy and aging). Surgical revision was performed in 12 cases (40%): in 3 patients it occurred after more than 5 years from previous breast reconstruction, and in 9 patients it was performed earlier (within the first 5 years after reconstruction). Important weight changes (e.g., weight loss more than 10 kg) occurred in 3 of these patients. Figure 2 shows the reconstructive journey of a 15-year-old patient with T2B1N2 severe malformation.

**Fig. 3:**
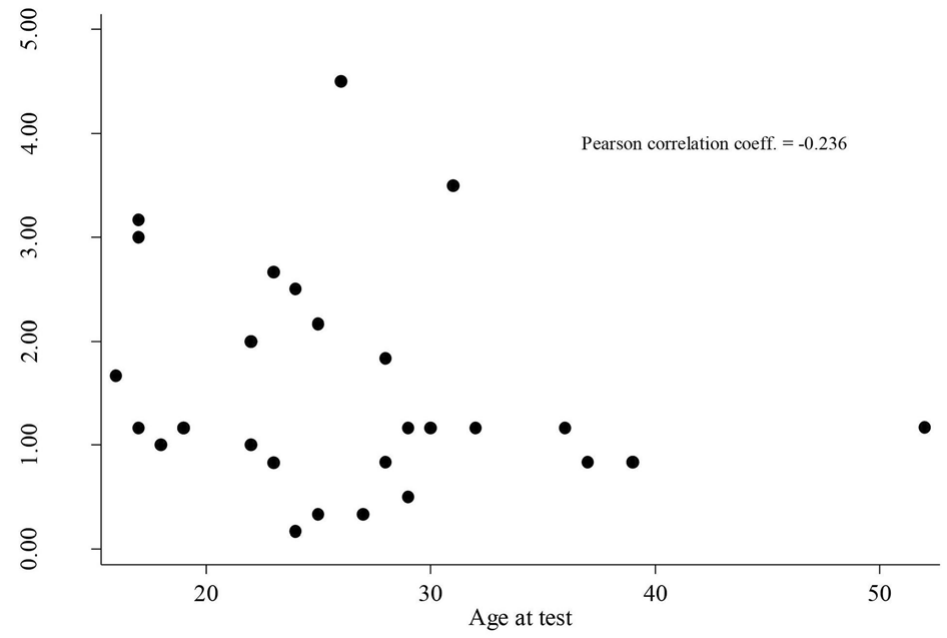
Correlation between CSM and age at questionnaire

Results deriving from the BUT questionnaire, divided by age group, are summarized in Table 2 and compared with general healthy population described by Cuzzolaro et al [13] in 2006. They show the absence of statistically significant differences in the severity scores related to the questionnaire, with the exception of Compulsive Self-Monitoring BUT-A subscale (CSM), for 16–17-year age group. Moreover, CSM appears to be related, even if weakly, with the age at the completion of the test: it seems to have a tendency to increase as age decreased (Figure 3).

**Table 2:** Body uneasiness test’s results.

Body uneasiness test						
	Poland syndrome patients 16–17 yr	General population 16–17 yr		Poland syndrome patients 18–39 yr	General population 18–39 yr	
	No. 4			No. 25		
	Mean ± sd	Mean ± sd	*p*	Mean ± sd	Mean ± sd	*p*
*BUT-A*						
GSI	1,60 ± 0,86	1.31 ± 0.78	0,459	1,36 ± 0,93	1.32 ± 0.91	0,830
WP	2,47 ± 1,22	1.97 ± 1.17	0,395	2,02 ± 1,26	1.92 ± 1.21	0,686
BIC	1,44 ± 0,81	1.65 ± 0.94	0,656	1,68 ± 1,15	1.62 ± 1.19	0,805
A	0,71 ± 0,72	0.61 ± 0.86	0,817	0,57 ± 0,63	0.56 ± 0.85	0,954
CSM	2,25 ± 0,99	1.37 ± 0.85	0,040	1,43 ± 1,02	1.61 ± 1.01	0,383
D	0,80 ± 0,99	0.64 ± 0.98	0,745	0,59 ± 1,04	0.84 ± 1.12	0,273

Data are obtained by comparing our results with Cuzzolaro M, Vetrone G, Marano G, Garfinkel PE. (2006) The Body Uneasiness Test (BUT): development and validation of a new body image assessment scale. Eat Weight Disord. 11: 1–13.

No statistically significant difference of BUT-A (GSI, A, WP, BIC, CSM and D) was found in relation to BMI, number of surgeries, age of beginning of the reconstructive process, association with hand and/or chest malformations, and onset of complications.

Results derived from BUT-B indicated a correlation between detachment and estrangement feelings toward one’s own body (*D*) and concerns about thighs and legs. On average, as a unit increases for “thighs”, “*D*” increases by 0.55 (*p* < 0.001) while as a unit increases for “legs”, “*D*” increases by 0.51 (*p* = 0.004).

Results deriving from the completion of the BREAST-Q questionnaire showed that satisfaction with breast (SB) resulted in an average percentage of 79.1% (range 45.8–94.4%), satisfaction with outcome of breast surgery (SO) was 94.9% (range 80.9–100%), psychosocial well-being (PW) was 78.5% (range 30–100%), sexual well-being (SW) was 75.3% (range 30–96.7%), and relative physical well-being in the chest and upper body (PWC) was 36.9% (20–68.7%).

The analysis of the results of the BREAST-Q questionnaire in relation to the BUT-*A* scores showed that high values of body uneasiness (GSI, A, BIC, D, WP) are significantly correlated with lower PW scores (*p *= 0.002, *p *= 0.002, *p *= 0.010, *p *= 0.014, *p *= 0.025). Moreover, avoidance behavior (*A*) is significantly correlated with lower SW scores (*p* = 0.037).

## Discussion

Breast abnormalities include a large group of anomalies, which can be quite frequent or very rare. Poland syndrome occurs in 1:20,000/30,000 both male and female live births, and it is generally associated to variable chest, subcutaneous tissue and skin anomalies that often make breast anomaly very complex [2]. A customized approach for each patient is mandatory through an in-depth preoperative analysis of patient expectations and careful explanation about limits of breast reconstruction procedures [17, 18]. Breast asymmetry in PS, in fact, is often classifiable as moderate (Grade *B*) and severe (Grade *C*) according to Persichetti’s classification of volume asymmetry [19], with the affected breast side really hypoplastic (hypomastia) or even absent (amastia). As described in the literature, these types of asymmetries require different approaches on each breast and are characterized by high incidence of secondary procedures [20]. In our series, 40% of patients required surgical revision in particular caused by body changes typical of the adolescent period or due to weight variations. Choosing to delay breast reconstruction in adulthood could have reduced surgical revisions but would have prolonged the discomfort due to malformations which, in adolescence, adds to frequently high general body dissatisfaction, especially widespread among girls [21–23] that, in developed countries, ranges between 35 and 81% and may be associated with unhealthy behaviors and psychosocial morbidities [24]. Moreover, when severe developmental distortions of breast size and shape are present, negative physical and emotional effects, such as early defensive behaviors, especially in body-exhibiting social situations, could affect women’s quality of life at any age [25–28].

Many approaches have emerged to address PS. In our center, the latissimus dorsi (LD) pedicled flap has been the standard of surgical care for PS patients for a long time [29] but we have over the years developed the conviction that the latissimus dorsi cannot compensate for the function of the pectoralis major muscle and thus we advise a more conservative approach mainly focused on the aesthetic purpose of recreating the breast mound [30]. We excluded from this study the patients who underwent reconstruction with flaps in order to avoid a possible bias in the results due to the impact of the functional impairment caused by the LD transposition [31].

Body uneasiness observed in our series of reconstructed patients is similar to that of a peer healthy female population [14]. We can therefore assume that breast reconstruction allows affected patients to have a quality of life comparable to healthy women of the same age group.

In those patients in whom body dissatisfaction was present after surgery, we found a general reduction in confidence in social settings; in particular, low SW was linked to the constant attention not to show one’s own body in public. Surprisingly, body dissatisfaction did not seem related to the severity of malformations and/or complexity of the surgical path. Moreover, specific concerns regarding body parts did not include body parts such as “chest” and “breast”. Only concerns for “thighs” and “legs” resulted in correlation to the presence of depersonalization symptoms, which generally occur starting from adolescence and are linked to emotional trauma in childhood [22]. Finally, youngest patients showed more frequently the tendency to continuously monitor their body, examining perceived defects and comparing oneself with peers even for many hours a day. This is in line with studies about girls, their dissatisfaction with at least 2 features of their body and their desire to be thinner [32].

Given the strong connections between body image and psychological well-being in women and girls [33–37], our findings highlight the importance of reconstructive surgery in female patients affected by PS. Many studies have found that breast remodeling surgery in case of asymmetry, tuberous breast [20] or macromastia [35] when psychosocial symptoms are present improves postoperative quality of life in both adults and adolescents [38]. In particular, postoperative BREAST-Q scores of our case series seem not to differ from average scores present in the literature. Only “physical well-being in the chest and upper body” resulted in a low average score, probably related to the presence of malformations of the upper limb and the rib cage, typical of Poland syndrome.

Finally, clinically significant body uneasiness in the preoperative period could affect postoperative satisfaction [39] up to even contraindicate surgery [40]. Identifying the presence of high level of clinical body uneasiness before surgery could be useful to program cognitive behavioral therapies to support patients during the reconstructive path and guarantee the best possible quality of life after breast reconstruction.

## Limitation of the Study

Poland syndrome is a rare disease, and therefore, the sample size is quite small. Furthermore, a preoperative evaluation of patients would have been useful to evaluate the improvement/worsening rate during the reconstructive process.

## Conclusions

Female patients affected by PS have to deal with complex breast and chest anomalies that can undermine quality of life. Breast reconstruction is generally able to improve psychosocial and sexual well-being linked to satisfaction with the appearance of the breast. However, general dissatisfaction with one’s own body, frequently present in females, could partly affect satisfaction with the final result.
